# Perspectives and Research Challenges in Veterinary Infectious Diseases

**DOI:** 10.3389/fvets.2014.00021

**Published:** 2014-11-10

**Authors:** Michael H. Kogut

**Affiliations:** ^1^Plains Agricultural Research Center, United States Department of Agriculture – Agricultural Research Service, College Station, TX, USA

**Keywords:** systems biology, innate immunity, patterns of pathogenesis, microRNA, metabolomics, proteomics and functional genomics

Technical breakthroughs have highlighted the host–microbe interactome to explore the immune system and the role for microorganisms in the development and function of the immune system. While these interaction studies have provided a window on the pathogenesis of the disease, there is a distinct lack of information on the effect of these interactions in the overall host physiology. For example, the seminal work conducted in human medicine demonstrating that subclinical inflammation has dramatic effects on host physiology resulting in diseases such as obesity, type 2 diabetes, and cardiovascular disease. This is an area of interest for this journal. What effects do subclinical infections have on the pathophysiology of animals resulting in what is believed to be acceptable production losses, but may actually be preventable?

The immune response and nutrient metabolism are two fundamental biological systems indispensable to maintaining and preserving life. Each of these systems is capable of modulating the activity of the other to ensure that the host animal is capable of coordinating the appropriate responses under any conditions. Thus, metabolic systems are integrated with pathogen-sensing and immune responses, and these pathways are evolutionarily conserved. Yet, we know very little about the effect of infections on host metabolism. Several important networks sense and manage nutrients and integrate with immune and inflammatory pathways to influence the physiological and pathological metabolic states. For example, the Toll-like receptors (TLRs), a family of the innate sensors, recognize specific microbial components, but can also sense nutritional signals, such as elevated glucose levels and saturated fatty acids. Likewise, metabolism-signaling pathways, such as leptin and other hormones, can also regulate immune functions. Thus, any immune alteration, specifically inflammation, can cause disturbances in host metabolism. New insights into infectious disease pathogenesis and animal production that can result in the discovery of novel diagnostic approaches and/or therapeutics are of high priority for the journal.

The Veterinary Infectious Disease specialty section seeks to become an outlet for veterinary research into infectious diseases through the study of the pathogen or its host or of the host’s environment, or by addressing combinations of these aspects of the disease system. We vision research in this area will exploit the very latest technologies to improve our understanding of the host–pathogen interface or its relationship with the host animal’s environment. With this in mind, we see the following areas of research that present needs and challenges in the immediate future.

## Systems Biology

The advancement of high-throughput technologies, together with the extensive identification of new genes, proteins, and other biomolecules in the “omics” era, has facilitated large-scale biological measurements. Cross-talk mechanisms during host–pathogen interactions impact the outcome of infections and further influence subsequent pathogen exposure. As a result, genome-wide studies provide global response patterns to infections from both the host and pathogen side. However, biological interpretations of genome-wide studies are limited to only a fraction of the theoretically possible interactions between genes, environmental conditions, and life cycles taking part in a host–pathogen setting. The enormous complexity underlying the host–pathogen interplay when considering the theoretically possible genetic interactions of even a few genes, necessitates the simplification of systems to cellular or pathway levels. A systems biology approach at the genomic, proteomic, and metabolomic levels is a continually emerging paradigm to better understand the pathophysiology of infectious processes and their underlying mechanisms during host–pathogen interactions. Systems biology connects many disciplines to create a quantitative and predictive interpretation of biological processes.

### Proteomics

For the most part, veterinary infectious disease research has focused on DNA analyses. Yet, the execution of the genetic plan is carried out by the activities of proteins. Gene transcription does not necessarily mean translation; mRNA can be silenced or destroyed before protein can be produced. In addition, proper folding, post-translational modifications, and activation are central to the proper functioning of a protein. Post-translational modifications such as phosphorylation and glycosylation are often extremely important for the function of many proteins, although most of these modifications cannot yet be predicted from genomic or mRNA sequences. Therefore, effective analytical techniques for quantitative determination of levels of protein phosphorylation are therefore essential tools for studies on the proteome, particularly in relation to infectious disease interactions, vaccine development, and drug discovery. For example, large-scale identification of phosphoproteins is possible due to advancements in methods of shotgun proteomics based on mass spectrometry. On the downside, these techniques require expensive instrumentation and involve complicated procedures for sample preparation through enrichment of phosphopeptides by enzymatic digestion. Microarray techniques involving peptide or protein arrays have demonstrated considerable potential as cost-effective, high-throughput, and convenient approaches for defining activities relating to signal transduction by means of phosphorylation reactions in a host have been implicated in a number of infectious human diseases (1–6). Further, using peptide arrays for kinome analysis is a new technique to study host cellular signaling during an infection. Kinome arrays facilitate parallel analysis of kinase activities and by comparison of treatments with and without a pathogen, one can detect kinases that become activated and deactivated upon infection (7). In addition, the use of species-specific peptide arrays for kinomic study provide further evidence for understanding mechanisms for host behavior during infection and can contribute to the rational design of more effective therapeutics and/or vaccines (8). Another under-utilized technique for use in veterinary infectious disease proteomics is proteome microarray analysis. Proteome microarrays provide a way to map the humoral responses to bacterial and viral proteomes; thus identifying proteins that could induce protective immune responses. These “immune signatures” have recently been shown to be used to predict vaccine efficacy (9) and design subunit vaccines for medically important viral and bacterial infections, respectively. Further, glycomic arrays are proteomic tools that are based on the carbohydrate binding specificities of proteins. Carbohydrates are recognized by viruses to invade host cells, whereas bacteria bind them to adhere to host cells.

### Metabolomics

Metabolomics provides a direct indication of the physiological status of a host during interactions with a pathogen by characterizing metabolites produced during an infection. The metabolic signature of the infected host represents the shared events of both host and pathogen and their metabolic crosstalk. Metabolomics uses analytical techniques to simultaneously analyze hundreds to thousands of small-molecule metabolites in biological samples. In conjunction with bioinformatics tools, metabolomics should help explain and predict clinical indicators of infections, host responses, disease pathogenesis/progression, and disease outcomes. Characterizing metabolic pathways driven by host–pathogen interaction can be achieved by profiling the downstream metabolites regulated indirectly by upstream genes. Unfortunately, a general lack of information of pathogen metabolism due to an absence of functional annotation for a large number of genes controlling regulatory metabolic enzymes may be the limiting factor for rapid progress of metabolomics in veterinary sciences. Thus, a great deal of annotation and functional genomics research is required before widespread adaptation of this omics approach can be widely utilized in veterinary infectious disease.

### miRNAomics

Medical research in infectious diseases and immunity has been shown to be profoundly influenced by microRNA (miRNAs). MiRNAs target and regulate several regulatory mRNAs that affect cellular activities including differentiation of multiple immune cells, regulation of multiple pattern recognition receptors (PRR)/NF-κB inflammatory signaling pathways in response to infections, and host cell activities that affect viral, bacterial, and parasitic colonization, persistence, and pathogenesis. Various miRNAs that can be induced or dysregulated during growth, development, and immune function have been found in the genomes of most farm and companion animals. An immediate research goal is to elucidate their functions in cellular processes and veterinary infectious diseases that would provide a better understanding of the molecular interactions between host and pathogen. For example, several human viruses encode miRNAs that down-regulate the expression of the functional activities of innate immunity, thus evading the host defenses. Additionally, many of these viruses also possess miRNAs to maintain latent and persistent infections. Do animal viruses also possess the miRNAs and do they function similarly? Likewise, what changes in miRNA expression are induced in the host cells and tissues of farm and companion animals colonized by pathogenic bacteria and what role do any changes in miRNA profiles have in either repressing or enhancing bacterial replication and persistence? Answering these questions can lead to the development of miRNA-based therapeutic approaches for the treatment of infectious diseases through either the restoration or repression of pathogen-specific miRNA expression and activity.

## Innate Immunity

The host immune response to pathogens in the earliest stages of infection is a critical determinant of disease resistance and susceptibility. These early responses are mediated by the innate immune system, a rapidly induced, phylogenetically conserved response of all multicellular organisms that depends upon a collection of germ-line-encoded PRRs for detection of microbe-associated molecular patterns (MAMPs) on or in major groups of microbes (10) or damage-associated molecular patterns (DAMPs). Sensing of MAMPs or DAMPs induces various extracellular activation cascades and intracellular signaling pathways, leading to the inflammatory response, recruitment of phagocytic cells for clearance of the pathogens, and mobilization of professional antigen-presenting cells.

Recent research in veterinary and medical infectious diseases have identified and characterized common and species-specific PRRs, signaling cascades, gene expression changes, and production of a range of host defense and inflammatory factors. The most studied and characterized family of innate receptors in veterinary species is the TLRs although viral research has also revealed the presence of the retinoic-acid inducible gene-I (RIG-I)-like receptors (viral RNA) as well. Fungal disease research has also revealed the presence of membrane bound C-type lectin receptors, which recognize mannan and β-glucan molecules. However, in general, there has been a lack of reports detailing the presence and function of several classes of cytosolic PRRs. The NOD-like receptors (NLR) and PYHIN protein family bind bacterial and viral moieties and form a cytoplasmic signaling complex known as the inflammasome that initiates activation of the protease precursor procaspase 1 to cleave proIL-1β and proIL-18 prior to their secretion as active inflammatory cytokines. The challenge here is to identify whether food and companion animals have these PRRs, characterize the pathogens and ligands that lead to inflammasome assembly, and what role they play in host defense against infections. Obviously, identification of inflammasomes and their functions can provide new targets for novel therapeutics in veterinary medicine.

Two other high priority areas of innate immunity research in veterinary infectious diseases that are lacking and could serve as the basis for future alternative therapeutics: (a) the emergence of “patterns of pathogenesis” as novel pathogen-induced inducers of innate immune responses and (b) modulation of innate immune system as an alternative to antimicrobials.

### Patterns of pathogenesis

The epithelial cells lining the mucosal barriers are a critical component of a communications network that is essential for transmitting signals generated in response to infection with microbial pathogens to cells of the innate and acquired immune systems. Because of these functions, the epithelium is considered as a “microbial sensor.” Like professional immune cells, epithelial cells recognize MAMPs and DAMPS to induce an innate response. However, the canonical PRR-MAMP (DAMP) cascades described above (TLR, NOD, RIG, C-type lectin) do not provide the epithelial cells with the discriminatory ability to differentiate between pathogenic and non-pathogenic microbes to which they are consistently exposed. It has been hypothesized that these cells require other sensors that can detect cellular perturbations or damage, which are triggered by pathogenic microbes but not commensals. It is these host cell perturbations that form the basis for a new concept on innate immune recognition and activation: patterns of pathogenesis (11). The patterns of pathogenesis concept expand innate recognition of MAMPs and sensing of DAMPS to include host detection of natural infection tactics used by pathogens to “infect, multiply within, and spread among their host” (11). These patterns would include growth (replication), cytosol access (pore formation and membrane damage), disruption of host cytoskeleton (secretion systems, toxins), and the newly identified pathogen-induced translation inhibition (12–14). These tactical infection patterns can then be used by non-professional immune cells to detect and to discriminate pathogenic and non-pathogenic microorganisms thereby directing specific immune responses. Identifying and/or confirming that such innate sensors exist veterinary species would be significant for pathogen diagnosis and development of future therapeutics and vaccines.

### Alternative to antimicrobials – modulation of innate immunity

Drug-resistant microorganisms pose an enormous threat to both veterinary and public health with an increasing rate of antimicrobial resistance being reported worldwide. The frequency of resistance is presumably due to extensive use of antibiotics as growth promoters in veterinary medicine has led to the emergence of multi-drug-resistant (MDR) strains that may pass from food animals to humans. In addition, fewer novel antibiotics are being developed to treat infectious diseases. Therefore, there is an increasing need to identify alternative approaches associated with less resistance. Current antimicrobial therapies target pathogen viability; while this approach is effective at eliminating the infectious agent, long-term treatment selects for resistant organisms. In addition to this increase in resistant organisms, normal flora beneficial to health can be adversely affected by antibiotic treatment. Alternative approaches for research should involve targeting (a) the host or host–pathogen interface rather than the pathogen and (b) facilitating pathogen-specific immune responses.

### Targeting the host–pathogen interface

A way to diminish the threat to human health would be to develop antimicrobial drugs that attack pathogens only without causing an imbalance in the normal bacterial flora. By using virulence factors as targets for novel anti-infectives, the probability for development of resistance may be low since resistance to compounds targeting the virulence factors cannot evolve and spread in the resident flora, as these bacteria lack virulence targets. We also propose that resistance to virulence-blocking agents is likely to result in non-functional virulence systems, and thus non-virulent microbes. Further, as long as the target remains, extracellular resistance cannot emerge through the activity of microbial efflux pumps. Finally, there will be a low selective pressure for mutations affecting the specific interaction between the drug and a virulence factor, as virulence blockers should have low effect on microbial growth.

### Targeting the host innate immune system

A fundamentally new strategy for the treatment of pathogens in food animals is to alter host immune responses to enhance the clearance of infectious agents and prevent or reduce tissue damage due to inflammation. Unlike conventional antibiotics that are designed to target a pathogen, modulating the immune system exert their protective effects by acting on the host. Vaccines are still the definitive immune-based prophylactic strategy for human and veterinary infectious agents. Yet, modern veterinary vaccines have multiple inadequacies including lack of cross-protection against several different strains of a pathogen, multi-antigenicity of pathogens, slow development of protective immunity (days versus hours), lack of adequate adjuvants, and a need for site-specific immune responses that require further advances in infection immunobiology to address these challenges and improve efficacy.

This is an exciting time to be conducting research in veterinary infectious diseases. The tools and technologies are becoming more available for use in veterinary species. It is my hope that the Section Veterinary Infectious Diseases in Frontiers in Veterinary Science becomes a primary outlet for the cutting edge research sure to come.

## Conflict of Interest Statement

The author declares that the research was conducted in the absence of any commercial or financial relationships that could be construed as a potential conflict of interest.

## References

[1] Lin M-H Hsu T-L Lin S-Y Pan Y-J Jan J-T Wang J-T Phosphoproteomics of *Klebsiella pneumonia* NTUH-K2044 reveals a tight link between tyrosine phosphorylation and virulence. Mol Cell Proteomics (2009) 8:2613–23. 10.1074/mcp.M900276-MCP200 19696081 PMC2816022

[2] Schmutz C Ahrne E Kasper CA Tschon T Sorg I Dreler RF Systems-level overview of host protein phosphorylation during *Shigella flexneri* infection revealed by phosphoproteomics. Mol Cell Proteomics (2013) 12:2952–68. 10.1074/mcp.M113.029918 23828894 PMC3790303

[3] Imani K Bhavsar AP Yu H Brown NF Rogers LD Finaly BB Global impact of *Salmonella* pathogenicity island 2-secreted effectors on the host phosphoproteome. Mol Cell Proteomics (2013) 12:1632–43. 10.1074/mcp.M112.026161 23459991 PMC3675819

[4] Nakayasu ES Tempel R Cambronne XA Petyuk VA Jones MB Gritsenko MA Comparative phosphoproteomics reveals components of host cell invasion and post-transcriptional regulation during *Francisella* infection. Mol Cell Proteomics (2013) 12:3297–309. 10.1074/mcp.M113.029850 23970565 PMC3820940

[5] Misra SK Milohanic E Ake F Mijakovic I Deutscher J Monnet V Analysis of the serine/threonine/tyrosine phosphoproteome of the pathogenic bacterium *Listeria monocytogenes* reveals phosphorylated proteins related to virulence. Proteomics (2011) 11:4155–65. 10.1002/pmic.201100259 21956863

[6] Rogers LD Brown NF Faang Y Pelech S Foster LJ. Phosphoproteomic analysis of *Salmonella*-infected cells identifies key kinase regulators and SopB-dependent host phosphorylation events. Sci Signal (2011) 4:rs9. 10.1126/scisignal.2001668 21934108

[7] Arsenault R Greibel P Napper S. Peptide arrays for kinome analysis: new opportunities and remaining challenges. Proteomics (2011) 11:4595–609. 10.1002/pmic.201100296 22002874

[8] Arsenault R Kogut MH Chicken-specific peptide arrays for kinome analysis: flight for the flightless. Curr Top Biotech (2013) 7:79–89.

[9] Legutki JB Johnston SA. Immunosignatures can predict vaccine efficacy. Proc Natl Acad Sci U S A (2013) 110:18614–9. 10.1073/pnas.1309390110 24167296 PMC3831987

[10] Janeway CA Medzhitov R Innate immune recognition. Ann Rev Immunol (2002) 20:197–216. 10.1146/annurev.immunol.20.083001.084359 11861602

[11] Vance RE Isberg RR Portnoy DA. Patterns of pathogenesis: discrimination of pathogenic and non-pathogenic microbes by the innate immune system. Cell Host Microbe (2009) 6:10–21. 10.1016/j.chom.2009.06.007 19616762 PMC2777727

[12] Fontana MF Banga S Barry KC Shen X Tan Y Luo Z-Q Secreted bacterial effectors that inhibit host protein synthesis are critical for induction of the innate immune response to virulent *Legionella pneumophilia*. PLoS Pathog (2011) 7:e1001289. 10.1371/journal.ppat.1001289 21390206 PMC3040669

[13] Dunbar TL Yan Z Balla KM Smelkinson MG Troemel ER. *C. elegans* detects pathogen-induced translational inhibition to activate immune signaling. Cell Host Microbe (2012) 11:375–86. 10.1016/j.chom.2012.02.008 22520465 PMC3334869

[14] Charkrabarti S Liehl P Buchon N Lemaitre B. Infection-induced host translational blockage inhibits immune responses and epithelial renewal in the *Drosophila* gut. Cell Host Microbe (2012) 12:60–70. 10.1016/j.chom.2012.06.001 22817988

